# H-reflex amplitude asymmetry is an earlier sign of nerve root involvement than latency in patients with S1 radiculopathy

**DOI:** 10.1186/1756-0500-4-102

**Published:** 2011-04-05

**Authors:** Hesham N Alrowayeh, Mohamed A Sabbahi

**Affiliations:** 1 Kuwait University, Faculty of Allied Health Sciences, Physical Therapy Department, State of Kuwait; 2 Texas Woman's University, School of Physical Therapy, Houston, Texas, USA

## Abstract

**Background:**

Based on our clinical experience, the H-reflex amplitude asymmetry might be an earlier sign of nerve root involvement than latency in patients with S1 radiculopathy. However, no data to support this assumption are available. The purpose of this study was to review and report the electrophysiological changes in H-reflex amplitude and latency in patients with radiculopathy in order to determine if there is any evidence to support the assumption that H-reflex amplitude is an earlier sign of nerve root involvement than latency.

**Results:**

Patients with radiculopathy showed significant amplitude asymmetry when compared with healthy controls. However, latency was not always significantly different between patients and healthy controls. These findings suggest nerve root axonal compromise that reduced reflex amplitude earlier than the latency parameter (demyelination) during the pathologic processes.

**Conclusion:**

Contrary to current clinical thought, H-reflex amplitude asymmetry is an earlier sign/parameter of nerve root involvement in patients with radiculopathy compared with latency.

## Background

The H-reflex is a useful electrophysiological procedure for diagnosing radiculopathy at the lumbosacral spinal level [1-7]. The recommended H-reflex diagnostic criteria are side-to-side latency differences [1,8], prolonged latency, absence of the H-reflex on the affected side [3,9,10], or H-reflex amplitude reduction on the affected side [2,4,7,8]. Other criteria include the threshold level of evoked potential and changes in the shape and number of phases of the H-reflex action potential. However, these criteria can be deceptive.

H-reflex latency prolongation or side-to-side differences in patients with radiculopathy probably indicates neural demyelination with significant damage of large diameter nerve axons [11]. Conversely, absent or reduced amplitude on the affected side is probably indicative of nerve conduction block in absence of extensive demyelination [12]. The conduction block decreases the recruitment of the spinal motoneurons [13], especially those fast conducting neurons.

Demyelination and conduction block may occur simultaneously and with varying degrees in radiculopathy [5,14]. However, in the acute stage, the H-reflex latency changes are less likely to be detected, especially when the neural impingement compromises the axonal function before enough demyelination has occurred. The effect of the changes in the H-reflex amplitude might be minimal, structurally and functionally, in the acute stage but may progress to full-fledged pathology with continuous neural impingement/compression. Thus, H-reflex amplitude changes (e.g., asymmetry, absence, reduction) may be more evident than latency changes (e.g., prolongation, side-to-side differences) in patients with early radiculopathy. With continued patterns of faulty posture and aggravation of the radiculopathy during functional daily activities, the severity of neural compromise at the root level may increase. As a result, changes in the H-reflex amplitude and latency will be more pronounced (i.e., absence of amplitude and/or latency prolongation or large side-to-side differences).

H-reflex amplitude, rather than latency, may be more evident in patients with radiculopathy as a result of nerve root compromise. However, no data to support this assumption are available. The purpose of this study was to review and report the electrophysiological changes in the soleus H-reflex amplitude and latency associated with the pathological processes in patients with lumbosacral radiculopathy at the S1 spinal level.

## Methods

### Participants

There were one hundred eighty participants in this study, including 55 healthy controls (30 males and 25 females between the ages of 18 and 60 years [mean (SD) = 39.69(12.4)]) and 125 patients (63 males and 62 females between the ages of 19 and 78 years [mean (SD) = 46.0 (13.4]). Participants read and signed an informed consent approved by the Ethical Review Board of Texas Woman's University.

Healthy controls were randomly selected from the university campus. None had any recent history of cancer, musculoskeletal, metabolic, systemic or neurologic disorders. Records of patients previously referred to our clinic for evaluation and treatment of lumbosacral radiculopathy were randomly reviewed. Patients had lumbosacral radiculopathy at S1 spinal level. The causes of radiculopathy were bulged disc, herniated disc or neuroforaminal stenosis. Radiculopathy was confirmed by the following criteria: clinical examination, imaging (MRI, CT, X-rays) and neurophysiologic studies.

The clinical criteria present with S1 radiculopathies were varying degrees of unilateral pain and paresthesia in the lumbosacral or lower limb, weakness in the triceps surae muscles and decreased Achilles tendon reflex [15]. The radiologic criteria for annular bulge used in this study were a broad-based extension of the disc beyond the body margins of the vertebral endplate, with smooth borders and a smooth internal nuclear signal. Herniation was seen as a focal defect with associated loss of the water contents of the nucleus and/or an irregular extension of the nuclear signal into the foramina. The EMG criteria were a unilateral denervation pattern in at least two muscles innervated by the affected nerve root as well as segmental paraspinal muscles, with no measurable changes in the sensory or motor conduction velocities.

Patients were excluded from the study if EMG reports showed bilateral peripheral nerve involvement, if H-reflex amplitude was absent (bilaterally), if there was recent history of musculoskeletal injury of the lower limbs (e.g., fracture, soft tissue lesion) and the spine (e.g., spine surgery, fracture), or if they had underlying systemic and metabolic disorders.

### H-reflex stimulation and recording

**Figure 1:**
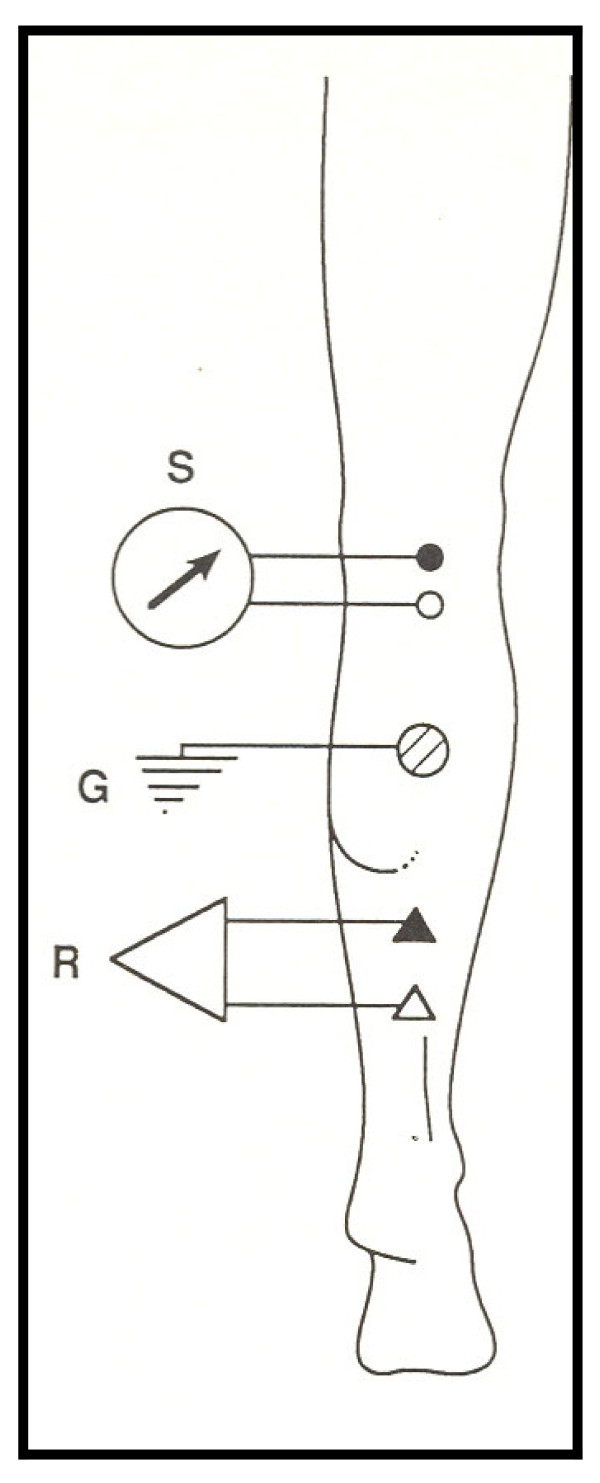
**Location of the stimulating and recording electrodes for the soleus H-reflex**. S = stimulus, R = recording, and G = ground electrodes.

With the participant lying prone, the soleus H-reflex was stimulated and recorded bilaterally, according to the method of Sabbahi and Khalil [5]. In brief, an EMG unit (Cadwell Lab., Kennewik, WA.) set at gain of 1000× to 5000× (1-5 mv./div.) and filter bandpass of 10 Hz-10 kHz elicited the soleus H-reflex by electrically stimulating the tibial nerve (duration = 1.0 ms, frequency = 0.2 PPS) at the popliteal fossa. The muscle response was then recorded using surface bar electrodes (Figure 1). A fixed distance was used between the stimulation and recording electrodes throughout the testing experiment (from popliteal fossa for stimulation to the recording electrodes at 3 cm. distal to the bifurcation of the gastrocnemii). The stimulation intensity for H-maximum was maintained by verifying the constant amplitude of the minimal M-wave. To control for the excitability of the motoneurones, participants were instructed to relax completely during data collection while keeping the head in the neutral position. This procedure reduced the reflex amplitude variability to the minimum. Seven to ten traces were elicited and recorded for each participant and the largest five traces were included in the analysis. The study procedures were approved by the Ethical Review Board of Texas Woman's University.

### Signal and data analyses

The peak-to-peak amplitude and latency to deflection of the five representative traces of the soleus H-reflex from the healthy controls and the patients were averaged. The side-to-side amplitude (H/H) ratios for the healthy controls were calculated according to the method of Jankus and colleagues [12] (i.e. smaller amplitude/larger amplitude). The patients' H/H ratios were calculated according to the method of Han and colleagues [8] (i.e. affected limb/non-affected limb). The affected limb was identified by positive EMG results. Based on Han et al study [8], side-to-side latency differences were used to divide the patients into two groups: 61 patients (30 males, 31 females) with normal latency (i.e., no side-to-side latency differences or differences smaller than 1.0 ms) and 64 patients (33 males, 31 females) with abnormal latency (i.e., side-to-side latency differences larger than 1.0 ms).

Descriptive statistics, including means and standard deviations for age, H-reflex amplitudes, H-reflex latencies, side-to-side H/H ratios, and side-to-side latency differences of all participants were calculated. One-way ANCOVA compared the statistical differences between the three groups (healthy controls, patients with normal latency, patients with abnormal latency) for H/H ratios and latency, with alpha = .05. Analysis of covariance was used to adjust for age difference between groups, and Bonferroni correction was used as post-hoc analysis. Using the Pearson correlation we examined the H-reflex with clinical and electrophysiological data.

## Results

All participants (healthy controls and patients) in this study showed an H-reflex. However, patients showed significant H-reflex amplitude asymmetry compared to healthy controls (*p *≤ .05; Tables 1). The H/H ratios were 0.67 and 0.54 in the patients with normal latency and patients with abnormal latency, respectively, and 0.83 in the healthy controls (Table 1). Post-hoc analysis using Bonferroni correction revealed that H/H ratios were significantly different between healthy controls and patients with normal latency (*p *= .04) and healthy controls and patients with abnormal latency (*p *= .0005). Latency was significantly longer in patients (*p *≤ .05; Table 1). Bonferroni correction showed latency was significantly different between healthy controls (latency = 29.98 ms; Table 1) and patients with abnormal latency (latency = 32.93 ms; Table 1) (*p *= .005). This was not the case between healthy controls (latency = 29.98 ms; Table 1) and patients with normal latency (latency = 31.44 ms; Table 1) (*p *= .33). These results suggest soleus H-reflex amplitude reduction on the affected side as a result of S1 nerve root compromise in patients with radiculopathy whether or not they have major latency changes. However, H-reflex amplitude asymmetry occurs earlier than latency changes in those patients with mild/acute radiculopathy (i.e., the duration of symptoms in patients with normal and abnormal latency was 31 and 57 days, respectively).

**Table 1:** Means and standard deviations for age, symptoms duration, soleus H-reflex amplitudes in millivolts (mV), soleus H-reflex latencies in millisecond (ms), soleus side-to-side amplitude H/H ratios, soleus side-to-side latency differences of all participants

		Healthy controls	Patients with normal latency	Patients with abnormal latency	
		Mean (SD)	Mean (SD)	Mean (SD)	*P-value*
**Age**		39.6 (12.4)	46.2 (12.3)	45.8 (14.4)	
**Amplitude (mV)**	Right or Symptomatic	5.98 (2.21)	3.95 (2.34)	2.37 (1.88)	
	Left or Asymptomatic	6.02 (2.03)	5.22 (2.75)	4.0 (2.34)	
**Latency (ms)**	Right or Symptomatic	29.98 (2.7)†	31.44 (2.69)	32.93 (2.82)†	.0005*
	Left or Asymptomatic	30.04 (2.6)	31.35 (2.72)	31.87 (2.74)	
**H/H ratio**		0.83 (0.11)‡§	0.67 (0.12)‡	0.54 (0.14)§	.0005*
**Latency differences**		0.12 (0.03)	0.31 (0.1)	1.36 (0.65)	
**Duration of symptoms (days)**		N/A	31.0 (8.6)	57.0 (8.2)††	

* Indicates significant differences (*p *< .05) for the soleus H-reflex parameters between groups using ANCOVA.

‡ Indicates significant differences (*p *< .05) between healthy controls and patients with normal latency for H/H ratio using Boferroni correction.

§ Indicates significant differences (*p *< .05) between healthy controls and patients with abnormal latency for H/H ratio using Boferroni correction.

† Indicates significant differences (*p *< .05) between healthy controls and patients with abnormal latency for latency parameter using Boferroni correction.

†† Indicates significant correlation between amplitude asymmetries and symptoms duration.

There was a strong correlation between amplitude asymmetries and the side of clinical symptoms (*r *= 0.7) and the duration of symptoms (*r *= 0.62) for patients with abnormal latency (Table 1). On the other hand, the correlation between amplitude asymmetries and the side of clinical symptoms and the duration of symptoms for patients with normal latency was weak (*r *= 0.01, *r *= 0.3; respectively).

## Discussion

Our results showed H-reflex amplitude asymmetry was more evident than latency changes in patients with radiculopathy. This was represented in the smaller value of the H-reflex H/H ratios whereas the latency differences were not always evident. We also noticed that changes in amplitude and latency were more associated with more chronic and/or severe cases of radiculopathy.

The reported H-reflex amplitude asymmetry and normal latency in patients with radiculopathy were probably due to conduction blockage in some large-diameter nerve axons [5,12,13]. Conduction blockage reduces the traveling neural signal in the nerve root [13]. It also results in desychronisation of input signal [16]. This decreased motoneuron recruitment and in amplitude reduction of descendant volley and H reflex [13,16]. As the severity (i.e., increase in the duration of symptoms and distal involvement of lower limbs pain, numbness, tingling, and weakness) of nerve root involvement progressed, the H-reflex amplitude asymmetry and abnormal latency were more pronounced. In this study, the H/H ratio was 0.67 when the latency was normal (duration of symptoms was 31 days, Table 1), and it reduced to 0.54 when the latency was abnormal (duration of symptoms was 57 days, Table 1). This indicates that as the pathology progresses and more neural axons are involved, changes (reduction) in H-reflex amplitude continues. It also suggests that H-reflex asymmetry is an early sign of neural compromise, even when the latency is normal.

Increased or prolonged latency in patients with neural impingement and LBP is a constantly reported finding [3,9,10], especially in more chronic conditions. Such latency change is not transient because it is caused by structural changes in the neural myelination (demyelination) as well as axonal damage, to a large extent. As these occur in chronic or long-standing conditions, a greater number of neural axons will be compromised and the patient's symptoms increase. Decompressing the nerve root, either mechanically or surgically, will not restore normal reflex latency. This might require more time for neural structural regeneration.

There is always concern among clinicians about using amplitude instead of the latency parameter for diagnostic purposes. This might be due to the high degree of reflex amplitude variability [17]. Reflex amplitude variability is the result of vestibular excitability, muscle activity, and cognitive state [18-21]. However, clinical and research-supported experience with the H-reflex shows that standardized testing will result in increased reflex stability [22]. Subject relaxation, use of taped-on stimulating electrodes and maintaining the head in the neutral position with arms symmetrically rested at the subject's sides improved reflex stability. It is also as important for the subject to empty his/her bladder before testing for increased reflex stability during the longer testing period. Repetitive stimulation at the beginning of the test and before collecting the data also results in stabilization of the tibial nerve threshold level to electrical stimulation, causing more stable H-reflex amplitude throughout the test [22]. These contributing effects would be less evident during standing (loading) compared with lying (unloading) [11].

Using the H-reflex, Jin et al [23] introduced a new sensitive measure to evaluate S1 radiculopathy. Specifically, they compared two methods of recording the H-reflex: conventional H-reflex and S1-foramen H-reflex. In the conventional H-reflex recording, the reflex is elicited by stimulation of the tibial nerve at the popliteal fossa, whereas in S1-foramen H-reflex, the H-reflex is elicited by stimulation of the S1 nerve root at the S1 foramen. In our study, only the conventional H-reflex was used to review and report the electrophysiological changes associated with the pathological processes in patients with lumbosacral radiculopathy at the S1 spinal level to support the assumption that H-reflex amplitude, compared to latency, may be an earlier indicator of nerve root involvement. This is particularly important because of inconsistency in published studies supporting H-reflex amplitude use. In the study by Jin etal. [23], ways to improve the sensitivity of H-reflex recordings were examined.

Several representative traces are important for clinical decision making. However, previous studies reported that four traces are the fewest needed to determine amplitude depression or recovery [24,25]. In this study, fives traces were recorded. Recording these traces is time consuming, especially if lower limbs are tested in lying and standing conditions [11]. However, the time involved is outweighed by the fact that the H-reflex amplitude is a more direct measure of changes in nerve root physiopathology that occurs in patients with radiculopathy.

The H-reflex amplitude and H/M ratio have long been used in the literature interchangeably. A previous study showed that both parameters relate the same information and behave similarly to intervention [11]. Another study recommended an H/H ratio smaller than 0.4 in the absence of latency differences, as measured in healthy individuals, to diagnose S1 neural involvement [12]. For patients with S1 radiculopathy, an H/H ratio smaller than 0.5 and a latency difference more than 1.0 ms was previously reported [8]. In this study, we found that an H/H ratio smaller than 0.67, in the absence of latency differences, indicated S1 neural involvement. Our results also emphasize the value of comparing both lower limbs rather than just reporting on the affected lower limb. Reflex asymmetry would, therefore, be the preferred parameter over a single data point.

## Limitation

A limitation of this study was the assumption made in the rationale for undertaking the study and the interpretation of findings. This assumption was based on our clinical experience with patients with radiculopathy which showed a preferential change in the H-reflex amplitude before latency parameter, given that the vestibular and supraspinal effects were controlled for. The electrophysiological findings reported in this study support this assumption. We did not correlate the degree of EMG changes or MRI findings to the degree of radiculopathy and symptomatic changes as this was not the objective of this study. However, these limitations are the focus of ongoing research. Another limitation of this study was the age difference between groups, although it was statistically controlled for. Given that the prevalence of neurological abnormal signs increases with age, differences between groups may be attributed to age differences. The findings were limited to the S1 nerve root involvement. Spinal nerve root compromises can be multisegmental, and this study did not reveal the diagnostic value of the H-reflex amplitude at other levels. Inter-subject differences is another limitation. Patients with bulged disc, herniated disc or foraminal stenosis had different degrees of neural compromise and the H-reflex parameters would be different in the three patients population. However, the main purpose of the study was to identify the most sensitive parameter in detecting radiculopathy and not the underlying cause of the pathology.

## Conclusion

H-reflex amplitude is a variable parameter whose diagnostic value has always been questioned. However, the results of this study support its use in the diagnosis of lumbosacral radiculopathy and suggest that comparative reflex amplitudes, such as the H/H ratio, might be preferred to the latency value as the dependent testing parameters. Thus, contrary to available clinical belief, H-reflex amplitude asymmetry compared with latency could be an earlier sign/parameter of nerve root involvement in patients with radiculopathy.

## Competing interests

We declare that we have no competing interests. Also, in the past five years we have not received reimbursements, fees, funding or salary from an organization that may in any way gain or lose financially from the publication of this manuscript, either now or in the future. We do not hold any stocks or shares in an organization that may in any way gain or lose financially from the publication of this manuscript, either now or in the future. Currently, we are not applying for any patents relating to the contents of the manuscript. We have not received reimbursements, fees, funding or salary from an organization that holds or has applied for patents relating to the contents of the manuscript.

## Authors' contributions

HNM and MAS equally contributed to the following activities: concept and research design, writing, project management, and data collection and analysis. All authors read and approved the final manuscript.
